# Perioperative Management Conundrum for a Case With Multiple Commonly Used Drug Hypersensitivity

**DOI:** 10.7759/cureus.53015

**Published:** 2024-01-26

**Authors:** Vikash Bansal, Habib Md R Karim, Abhishek K Rai, Dipak Bhuyan, Sanjay Kumar

**Affiliations:** 1 Anaesthesiology, Critical Care, and Pain Medicine, All India Institute of Medical Sciences, Deoghar, Jharkhand, IND; 2 Burn and Plastic Surgery, All India Institute of Medical Sciences, Deoghar, Jharkhand, IND

**Keywords:** mefenamic acid, diclofenac allergy, paracetamol allergy, anaesthesia and analgesia, drug hypersensitivity reactions

## Abstract

Perioperative hypersensitivity reactions vary from mild to potentially fatal anaphylaxis, resulting in significant morbidity and mortality. Most of the perioperative hypersensitivity and allergic reactions are attributed to antibiotics, antiseptic solutions, latex, and opioids. In the current thrust for opioid-free anesthesia, owing to its multiple advantages, paracetamol and nonsteroidal antiinflammatory agents play a significant role in multi-modal pain and inflammatory response management. Nearly nine out of ten individuals experience postoperative pain, one-third experience postoperative nausea and vomiting, and one-fourth experience fever, irrespective of surgery and type of anesthesia, often as an inflammatory response. While perioperative hypersensitivity reactions are common, a patient allergic to multiple commonly used drugs for the treatment of pain, fever, acid-peptic disorder, and nausea and vomiting is scarce. Such cases pose a great challenge in perioperative management. A 14-year-old male child with a traumatic foot drop planned for tibialis posterior tendon transfer developed an allergic reaction with mild fever following an injection of Ranitidine and Ondansetron in the preoperative area. Surgery was deferred and was investigated for allergy profile testing for commonly used drugs, which showed high IgE levels and moderate to severe hypersensitivity for diclofenac and paracetamol. The patient was operated on after one month under spinal anesthesia, avoiding ranitidine, ondansetron, diclofenac, and paracetamol. The following morning, he developed a high-grade fever (102.3° F), which did not resolve with conservative measures. Hypersensitivity and allergic reactions to NSAIDs are reported in the literature. While there are multiple drugs available as NSAIDs, cross-sensitivity or allergy to other drugs within the same group, and even chemically related groups, is also another possibility that needs to be considered while managing such patients. Mefenamic acid controlled the fever, and the child was discharged home after 48 hours of observation. However, the case posed a great perioperative management dilemma; the present report intends to highlight and discuss it.

## Introduction

Perioperative hypersensitivity reactions are frequent and crucial interdisciplinary issues that require a multidisciplinary approach. Most of these reactions are mild to moderate in intensity [1,2]. Nonetheless, they can even be severe, requiring immediate resuscitation and life support [1,3]. The most common drugs responsible for perioperative hypersensitivity and allergic reactions are antibiotics in the cephalosporin group (36.8%), antiseptics like chlorhexidine and betadine (21%), and opioids like morphine (13.1%) [2]. Latex allergy is also common in children [2]. In the current thrust for opioid-free anesthesia, owing to its multiple advantages, paracetamol and nonsteroidal antiinflammatory agents play a significant role in multi-modal pain and inflammatory response management.

Irrespective of the invasiveness of the surgical procedure performed, all surgical patients suffer some degree of pain and inflammation; 86% complain about postoperative pain, and analgesic and anti-inflammatory drugs are used to make the pain tolerable [4]. Nearly one-quarter of surgical patients experience postoperative fever, irrespective of surgery and type of anesthesia [5]. Postoperative nausea and vomiting (PONV) incidence within the first 24 hours also ranges from 28.7% (95% confidence interval 23.8 to 33.6) to 73.4% in selected high-risk groups [6,7]. Symptoms like pain and PONV are troublesome and impact the outcome and satisfaction [8]. The current standards and ideal planning for perioperative pain management use multimodal analgesia techniques and multimodal drug therapy for the prevention and control of PONV. Paracetamol, non-steroidal anti-inflammatory drugs (NSAIDs), and 5-HT blockers are crucial components of such treatments [9,10]. Therefore, a patient with an allergy to multiple medicines poses a tremendous perioperative challenge. Further, perioperative allergic reactions, which can even be life-threatening [1,3], are complicated to diagnose under anesthesia as patients cannot report the symptoms, many of the signs are masked, and the skin is under drape.

Hypersensitivity and allergic reactions to NSAIDs are reported in the literature. However, an allergy to multiple regularly used drugs in a single patient is rare [11]. Further, cross-sensitivity or allergy to other related medications, i.e., mefenamic, naproxen, and acetaminophen cross-sensitivity; penicillin and first-generation cephalosporin cross-allergy, is another possibility that needs to be considered while managing such cases. Such cross-sensitivity reactions can be immunologic or non-immunologically mediated [12]. The present report intends to highlight such a perioperative dilemma and discuss the possible management steps.

## Case presentation

A 14-year-old male child, average in build and height, was posted for correction of traumatic foot drop by tibialis posterior tendon transfer. He had no comorbidities. Except for a few over-the-counter analgesics, he has not consumed any drugs, food supplements, etc., in the past. Preoperative clinical findings were unremarkable for vitals and systemic examinations. On the morning of surgery, injections of Ondansetron 2 mg and Ranitidine 25 mg were administered intravenously (IV) at an interval of five minutes as per surgical team protocol. After five minutes, he complained of itching in his left arm, having the venecath in situ. On examination, rashes and urticaria involving the left arm and forearm were noted; vitals were pulse rate (PR) 110/min, blood pressure (BP) 108/72 mmHg, oxygen saturation (SpO2) 98% on room air, and respiratory rate (RR) 14/min. The child was fully conscious and oriented, with no difficulty breathing or maintaining the airway. Immediately, IV pheniramine (20 mg) and hydrocortisone (100 mg) were administered; Ringer's lactate was started. Surgery was deferred due to an unanticipated hyperacute allergic reaction, as the nature of the surgery was elective. He also developed a mild fever.

An IgE level and drug allergy profile for commonly used medications were advised. IgE level was 578 IU/mL (normal range 1.90-170) and showed severe allergic potential to paracetamol and diclofenac (Annexure 1). Table 1 shows the allergic potential for common analgesics, anti-inflammatories, and antibiotics used and tested. The patient was scheduled for surgery after one month. During pre-anesthesia check-ups, avoiding the allergen drugs was advised. Further, tablets Levocetirizine 5 mg and Montelukast 10 mg were advised preoperatively for three days. Lignocaine skin sensitivity in the morning of surgery was also suggested, which showed indeterminate results; however, the allergic test result was suggestive of no risk (Table 1).

**Table 1:** Shows the allergic potential for common analgesics, anti-inflammatories, and antibiotics used and tested. All units are in IU/ml. The normal level for all allergens is ≤ 0.35 IU/ml. IU stands for international unit.

Common Anti-microbials				Common Analgesics	
Ampicillin	0.34	Norfloxacillin	0.05	Paracetamol	1.00
Cloxacillin	0.26	Tetracycline	0.08	Diclofenac	1.40
Penicillin	0.16	Ciprofloxacin	0.12	Brufen	0.18
Amoxycillin	0.08	Sulpha	0.29	Xylocaine	0.04
Cefixime	0.30	Doxycycline	0.03	Aspirin	0.24
Cefuroxime	0.02			Nimesulide	0.01

With informed consent and counseling, he was taken to the operation theater without any premedication; the American Society of Anesthesiologists standard monitoring was attached. Subarachnoid block (SAB) was performed with 0.5% bupivacaine (heavy) in 2.6 mL, and midazolam 0.5 mg IV was administered following SAB. Intraoperatively, the patient had an uneventful course except for shivering; tramadol 30 mg slow IV controlled it. Local infiltration with 12 mL of bupivacaine 0.25% was done, and tramadol was prescribed as a rescue analgesic.

Postoperatively, he was shifted to a high-dependency unit for close monitoring. On the evening of surgery (day 0), the patient developed a fever of 101.2° F, for which cold sponging was advised as he was allergic to paracetamol. Again, he had 102.3° F at night, not resolving with cold sponging techniques. However, the fever was persistent. So, after preparing all resuscitative measures, half a tablet of 250 mg of mefenamic acid was administered orally; the fever resolved to 98.2° F after an hour. IV fluid was maintained, and the patient was encouraged to take water.

**Figure 1:**
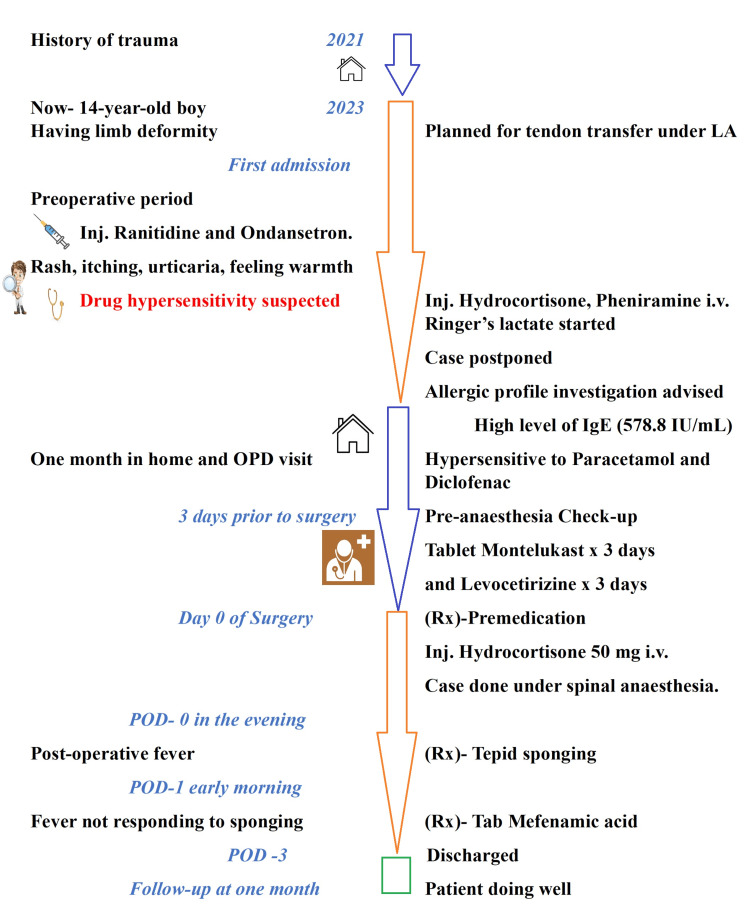
Case timeline and events. OPD: outpatient department, POD: post-operative day, LA: local anesthesia, IgE: immunoglobulin E, IU: international unit.

While measures were taken to decrease the fever, the cause of fever-like infections and any incidental allergic drug administration were evaluated. Complete blood counts, urine routine, malaria, typhoid, and dengue tests were sent. The total leucocyte counts (TLC) were 7,560/dL, with neutrophils comprising of 89.5%. Other investigations were within normal limits. The patient's pain control was adequate, and no further events were noted. He was discharged to home after 48 hours of observation. The case timeline is presented in Figure 1.

## Discussion

The present case posed a great dilemma as the surgery was elective and minor. The physical status of the child was also healthy, but he presented with a hyperacute allergic reaction to the use of common drugs used in the perioperative period. Lower limb surgery, even in children, can be done under SAB, which can reduce the use of multiple IV drugs required in general anesthesia (GA). Postoperative fever and pain can even happen after SAB, as these symptoms are infrequently due to anesthesia drugs or techniques. The intensity and outcome of perioperative hypersensitivity reactions can even be potentially fatal [13]. Therefore, taking such a high risk for elective minor or intermediate surgery without proper work-up and optimization raises good clinical practice concerns. Patient evaluation, including investigations, should be done for such reactions for preoperative optimization and perioperative anesthesia management to prevent significant side effects, and patients with a history of severe perioperative adverse effects or perioperative anaphylaxis that has not been formally evaluated should be sent to an allergy clinic [14]. The intraoperative period needs extra vigilance as surgical drapes cover the patient's skin, and rashes, urticaria, and flushing might be easily missed. Mild variations in PR and BP might not be interpreted as possible reactions to anesthesia, as such variations are routine intraoperatively. Itching and abdominal pain cannot be reported under GA or regional anesthesia (RA) plus sedation.

The mechanisms responsible for perioperative hypersensitivity and allergic reactions are multidimensional. In most cases, it is impossible to distinguish between an allergic or non-allergic underlying cause in clinical practice [15,16]. When symptoms and signs suggest allergic reactions, the patient is assumed to suffer from an allergic reaction until subsequent allergic investigations are negative [14-16]. Ruling out anesthetic or surgical causes and the pharmacological effect of any drug from the involvement of the immune system or inflammatory mechanisms is imperative [14,15]. Allergic reactions are characterized by the specific activation of IgE antibodies and, rarely, IgG antibodies. On the other hand, the non-allergic inflammatory mechanism includes activating the cyclooxygenase pathway, the Kinin-Kallikrein system, non-specific mast cells, basophil mediator release, etc. IgE and tryptase-level evaluation might help in differentiating them [15,16]. Our patient presented with immediate skin and mild systemic inflammatory signs and symptoms on the first hospitalization following the administration of ranitidine and ondansetron. Subsequently, the IgE evaluation showed a significantly raised level. However, we could not do specific IgE and tryptase levels to differentiate the reaction type due to financial constraints.

Allergic testing of the present case indicated severe potential for paracetamol and diclofenac, i.e., >1 IU/ml. On the other hand, the patient showed allergic manifestations following ranitidine and ondansetron administration at the previous admission. The lignocaine skin test was also indeterminate. All these drugs are frequently required for an acid-peptic disorder, gastro-esophageal reflux, PONV, fever, and pain management in the perioperative period. So, multi-drug allergies pose a significant challenge in such a situation. Further, cross-sensitivity for other similar drugs also cannot be denied, and it is not viable to test each possible drug in developing and third-world countries where the facilities and cost become a hindrance. In one-quarter of cases, cause determination for the allergic reaction is impossible, and an adverse event might have been mistakenly diagnosed as anaphylaxis [13]. Clear labeling, a bedside note alerting the patient to the allergy, and providing information on which substitute product to use in case of accidental re-exposure are effective ways to prevent unintentional re-exposure.

The mainstay of the allergic reaction in the perioperative period is epinephrine and volume expansion with intravenous fluid [17,18]. However, epinephrine use is mostly limited to moderate to severe reactions with cardiovascular and respiratory signs and symptoms. Pre-treatment with histamine-receptor antagonists or corticosteroids lacks strong evidence of reducing the severity of anaphylaxis [15,17,18]. Nevertheless, in patients with recurring reactions caused by non-specific histamine release, premedication with antihistamines may be beneficial [14,15]. Although no studies have proven the benefit of using antihistamine chlorpheniramine in perioperative anaphylaxis, NAP6 results show no harm from its use [14]. Therefore, these can be advised to reduce or prevent such reactions. Intravenous corticosteroids may be given after adequate resuscitation. In our patient, the preoperative use of mast-cell stabilizer, steroid, and antihistamine was intended to reduce the chance of anaphylactic reaction in cases with mastocytosis [19].

Further, all resuscitation procedures were kept ready. RA is favored since it lowers the chance of GA-related polypharmacy, especially the need for muscle relaxants [13,14]. Further, local anesthetics used in the RA also have anti-inflammatory actions. Therefore, a tailored approach is required, and the patient's level of stabilization and surgical urgency should determine whether and how to proceed.

A fever that develops within hours on the operative day is usually alarming, as it might result from malignant hyperthermia and sepsis. First- or second-day postoperative fever is typically related to non-infective inflammatory responses, and diagnostic testing is unnecessary unless there are concurrent symptoms [5,20]. An inflammatory reaction to surgical stress most likely caused POD-1 fever in our patient because all other examinations came back normal, and the elevated neutrophil counts, along with regular TLC, suggested a non-infectious inflammatory response, which resolved after giving mefenamic acid. Notably, mefenamic acid also acts as an anti-inflammatory. Kulkarni SJ et al. also reported a case of allergy to paracetamol and diclofenac and not to ibuprofen [11]. Our testing also showed the same. Diclofenac, ibuprofen, and mefenamic acid are non-specific on the COX receptor but are structurally different; diclofenac and ibuprofen, which belong to the heteroaryl acetic acid group, have a higher incidence of anaphylactic reactions [21].

Further, allergy testing is unreliable for 6-8 weeks following an allergic reaction [13]. An oral provocative test (OPT) or skin patch to find a safe drug can be performed, but such tests can also provide contradictory results, and NSAID cross-reactivity cannot be ascertained [22]. Nevertheless, OPT can also produce significant symptoms and signs. Therefore, we used mefenamic acid, which has shown a similar effect to high-dose paracetamol in children [23].

Our case report is, however, limited by the fact that we could not categorize it specifically by the type of reaction it was or which exact agent was the triggering factor. Most of the perioperative hypersensitivity and allergic reactions present immediately within a few minutes of administering the triggering agents. Raised IgE also hinted toward a possible type-I reaction. Yet, cutaneous changes can even happen in non-allergic reactions. Further, delayed hypersensitivity to medications administered in the preoperative period and manifesting in the postoperative period is also a concern [24]. 

## Conclusions

Commonly used poly-drug hypersensitivity poses a significant challenge during the perioperative period, even for minor and intermediate surgeries. Even the RA technique cannot avoid polypharmacy, as pain, fever, and PONV are frequent happenings. Mefenamic acid might be a safe anti-inflammatory, antipyretic, and analgesic. Ibuprofen is another agent to consider, especially in children. Allergic testing for commonly used drugs can be helpful in elective cases. However, such tests have limitations and poor reliability if performed within a few weeks of experiencing a hypersensitivity reaction, and all resuscitative measures must be kept at the bedside while managing such patients.

## Figures and Tables

**Figure 2:**
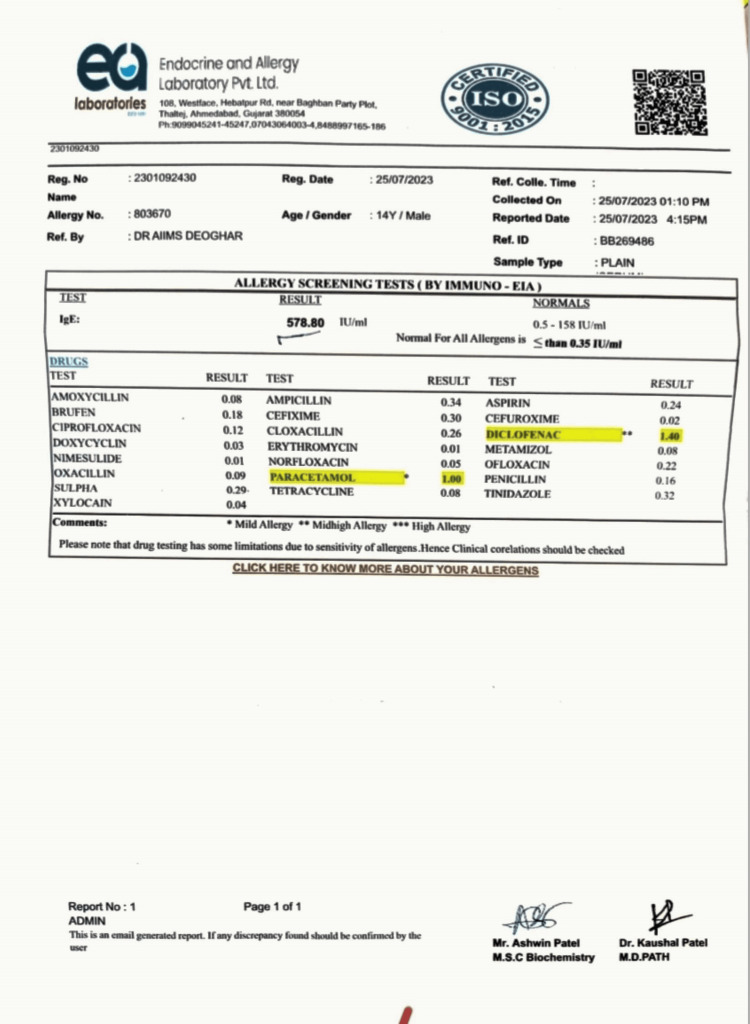
Scan copy of the allergic profile analysis report.

## References

[1] Kosciuczuk U, Knapp P (2021). What do we know about perioperative hypersensitivity reactions and what can we do to improve perioperative safety?. Ann Med.

[2] Krikeerati T, Wongsa C, Thongngarm T (2023). Perioperative immediate hypersensitivity incidence, clinical characteristics, and outcomes after allergological evaluation: A multi-disciplinary protocol from tertiary hospital, Thailand. Asian Pac J Allergy Immunol.

[3] Poonuraparampil JA, Karim HR, Garhwal A, Babu MJ (2020). The conundrum of perioperative management for emergency cesarean section in a patient with anaphylactic shock. Bali J Anesthesiol.

[4] Gan TJ, Habib AS, Miller TE, White W, Apfelbaum JL (2014). Incidence, patient satisfaction, and perceptions of post-surgical pain: results from a US national survey. Curr Med Res Opin.

[5] Lesperance R, Lehman R, Lesperance K, Cronk D, Martin M (2011). Early postoperative fever and the "routine" fever work-up: results of a prospective study. J Surg Res.

[6] Amirshahi M, Behnamfar N, Badakhsh M, Rafiemanesh H, Keikhaie KR, Sheyback M, Sari M (2020). Prevalence of postoperative nausea and vomiting: A systematic review and meta-analysis. Saudi J Anaesth.

[7] Adámek S, Matoušková O, Polanecký O, Světlík S, Skořepa J, Šnajdauf M (2013). The effect of postoperative pain treatment on the incidence of anastomotic insufficiency after rectal and rectosigmoideal surgery. Prague Med Rep.

[8] Goth A, Karim HM, Yunus M, Chakraborty R, Dey S, Bhattacharyya P (2023). Effect of postoperative anesthesiologists' single visit on patient satisfaction: a hospital-based non-randomized study. Cureus.

[9] Schwenk ES, Mariano ER (2018). Designing the ideal perioperative pain management plan starts with multimodal analgesia. Korean J Anesthesiol.

[10] Gan TJ, Belani KG, Bergese S (2020). Fourth consensus guidelines for the management of postoperative nausea and vomiting. Anesth Analg.

[11] Kulkarni SJ, Kelkar VP, Nayak PP (2014). Anesthesia in a patient with multiple allergies. J Anaesthesiol Clin Pharmacol.

[12] Romano A, Guéant-Rodriguez RM, Viola M, Gaeta F, Caruso C, Guéant JL (2005). Cross-reactivity among drugs: clinical problems. Toxicology.

[13] Savic L, Stannard N, Farooque S (2020). Allergy and anaesthesia: managing the risk. BJA Educ.

[14] Harper NJ, Cook TM, Garcez T (2018). Anaesthesia, surgery, and life-threatening allergic reactions: management and outcomes in the 6th National Audit Project (NAP6). Br J Anaesth.

[15] Garvey LH, Dewachter P, Hepner DL (2019). Management of suspected immediate perioperative allergic reactions: an international overview and consensus recommendations. Br J Anaesth.

[16] Volcheck GW, Melchiors BB, Farooque S (2023). Perioperative hypersensitivity evaluation and management: a practical approach. J Allergy Clin Immunol Pract.

[17] Shaker MS, Wallace DV, Golden DB (2020). Anaphylaxis-a 2020 practice parameter update, systematic review, and Grading of Recommendations, Assessment, Development and Evaluation (GRADE) analysis. J Allergy Clin Immunol.

[18] Ring J, Beyer K, Biedermann T (2014). Guideline for acute therapy and management of anaphylaxis: S2 Guideline of the German Society for Allergology and Clinical Immunology (DGAKI), the Association of German Allergologists (AeDA), the Society of Pediatric Allergy and Environmental Medicine (GPA), the German Academy of Allergology and Environmental Medicine (DAAU), the German Professional Association of Pediatricians (BVKJ), the Austrian Society for Allergology and Immunology (ÖGAI), the Swiss Society for Allergy and Immunology (SGAI), the German Society of Anaesthesiology and Intensive Care Medicine (DGAI), the German Society of Pharmacology (DGP), the German Society for Psychosomatic Medicine (DGPM), the German Working Group of Anaphylaxis Training and Education (AGATE) and the patient organization German Allergy and Asthma Association (DAAB). Allergo J Int.

[19] Lide B, Mcguire S, Liu H, Chandler C (2022). Mast cell activation syndrome-anesthetic challenges in two different clinical scenarios. J Biomed Res.

[20] O'Mara SK (2017). Management of postoperative fever in adult cardiac surgical patients. Dimens Crit Care Nurs.

[21] Quiralte J, Blanco C, Delgado J (2007). Challenge-based clinical patterns of 223 Spanish patients with non-steroidal anti-inflammatory-drug-induced-reactions. J Investig Allergol Clin Immunol.

[22] Ammar H, Ben Fredj N, Ben Romdhane H, Chaabane A, Chadli Z, Ben Fadhel N, Aouam K (2023). Cross-reactivity between nonsteroidal anti-inflammatory drugs in fixed drug eruption: Two unusual cases and a literature review. Br J Clin Pharmacol.

[23] Loya A, Siddiqui MS, Sangle A, Ingale V, Saha S, Nelanuthala M (2022). The antipyretic effect of high-dose Paracetamol versus mefenamic acid in the treatment of febrile children: a randomized control trial. Cureus.

[24] Chu EC, Huang KH, Cheung G, Ng G, Lin A (2023). Delayed skin allergy to glucosamine chondroitin supplement. Cureus.

